# Heterologous gene expression system for the production of hydrolyzable tannin intermediates in herbaceous model plants

**DOI:** 10.1007/s10265-023-01484-2

**Published:** 2023-08-01

**Authors:** Chihiro Oda-Yamamizo, Nobutaka Mitsuda, Carsten Milkowski, Hideyuki Ito, Kentaro Ezura, Ko Tahara

**Affiliations:** 1 Department of Forest Molecular Genetics and Biotechnology, Forestry and Forest Products Research Institute (FFPRI), 1 Matsunosato, Tsukuba, Ibaraki 305-8687 Japan; 2 Bioproduction Research Institute, National Institute of Advanced Industrial Science and Technology (AIST), Tsukuba, Ibaraki 305-8566 Japan; 3 Global Zero Emission Research Center, National Institute of Advanced Industrial Science and Technology (AIST), Tsukuba, Ibaraki 305-8566 Japan; 4 AGRIPOLY: International Graduate School in Agricultural and Polymer Sciences, Martin Luther University Halle-Wittenberg, Betty-Heimann-Straße 3, 06120 Halle, Germany; 5 Faculty of Health and Welfare Science, Okayama Prefectural University, 111 Kuboki, Soja, Okayama 719-1197 Japan; 6 Japan Society for the Promotion of Science, Tokyo, Japan

**Keywords:** Biosynthetic pathway, *Eucalyptus camaldulensis*, Herbaceous model plants, Heterologous expression, Hydrolyzable tannin

## Abstract

Aluminum toxicity is the main factor limiting the elongation of plant roots in acidic soil. The tree species *Eucalyptus camaldulensis* is considerably more resistant to aluminum than herbaceous model plants and crops. Hydrolyzable tannins (HTs) accumulating in *E. camaldulensis* roots can bind and detoxify the aluminum taken up by the roots. However, in herbaceous model plants, HTs do not accumulate and the genes involved in the HT biosynthetic pathway are largely unknown. The aim of this study was to establish a method for reconstituting the HT biosynthetic pathway in the HT non-accumulating model plant *Nicotiana benthamiana*. Four *E. camaldulensis* enzymes were transiently expressed in *N. benthamiana* leaves via *Agrobacterium tumefaciens*-mediated transformation. These enzymes included dehydroquinate dehydratase/shikimate dehydrogenases (EcDQD/SDH2 and EcDQD/SDH3), which catalyze the synthesis of gallic acid, the first intermediate of the HT biosynthetic pathway that branches off from the shikimate pathway. The others were UDP-glycosyltransferases (UGT84A25 and UGT84A26), which catalyze the conversion of gallic acid to β-glucogallin, the second intermediate. The co-expression of the EcDQD/SDHs in transgenic *N. benthamiana* leaf regions promoted the synthesis of gallic acid*.* Moreover, the co-expression of the UGT84As in addition to the EcDQD/SDHs resulted in the biosynthesis of β-glucogallin, the universal metabolic precursor of HTs. Thus, we successfully reconstituted a portion of the HT biosynthetic pathway in HT non-accumulating *N. benthamiana* plants. This heterologous gene expression system will be useful for co-expressing candidate genes involved in downstream reactions in the HT biosynthetic pathway and for clarifying their in planta functions.

**Supplementary Information:**

The online version contains supplementary material available at 10.1007/s10265-023-01484-2.

## Introduction

Aluminum (Al) toxicity is a major abiotic stress limiting the productivity of plants growing in acidic soil, which covers approximately 30% of the total land area worldwide (von Uexküll and Mutert 1995). Under acidic conditions below pH 5, Al is released into the soil solution from minerals, mainly as Al^3+^, and the accumulation of Al in root tips rapidly inhibits root elongation, ultimately leading to decreased water and nutrient uptake (Kopittke et al. 2016; Ma 2007). Therefore, the Al resistance of crops and trees must be improved to ensure sustainable food and forest production. *Eucalyptus camaldulensis* is a tree species that can grow in acidic soil and show no inhibition of root elongation when exposed to millimolar levels of Al (Tahara et al. 2008). The Al resistance of *E. camaldulensis* is 200- to 1,000-times higher than that of herbaceous model plants and crops. We previously isolated a hydrolyzable tannin (HT), oenothein B, from *E. camaldulensis* roots as a novel Al-binding ligand, and determined that the binding of Al^3+^ by oenothein B contributes to the detoxification of Al entering *E. camaldulensis* roots (Tahara et al. 2014). Tannins are a mixture of polyphenols found in plant leaves, bark, and wood, with molecular weights ranging from 500 to more than 3,000; they are divided into two types according to their chemical structure and properties: HTs and condensed tannins (Hagerman and Buttler 1981; Hassanpour et al. 2011). Condensed tannins are present in many plant species, whereas HTs are considered to accumulate preferentially in angiosperm plants excluding monocots (Bate-Smith 1984). Hydrolyzable tannins are widely distributed among long-lived woody plants, but they are absent in herbaceous model plants. This has hindered the elucidation of HT biosynthesis and regulation. Additionally, HTs reportedly have diverse functions, including as defense-related compounds that protect plants from herbivorous insects (Agrawal et al. 2012; Barbehenn and Constabel 2011) and mammals (Takahashi and Shimada 2008) as well as from microbial pathogens (Buzzini et al. 2008). Moreover, our previous research on *E. camaldulensis* showed that HTs have ecologically important effects because they can detoxify toxic metals (Tahara et al. 2014; 2017; Zhang et al. 2016). To effectively exploit these biological functions, the HT biosynthetic genes must be identified.

**Fig. 1:**
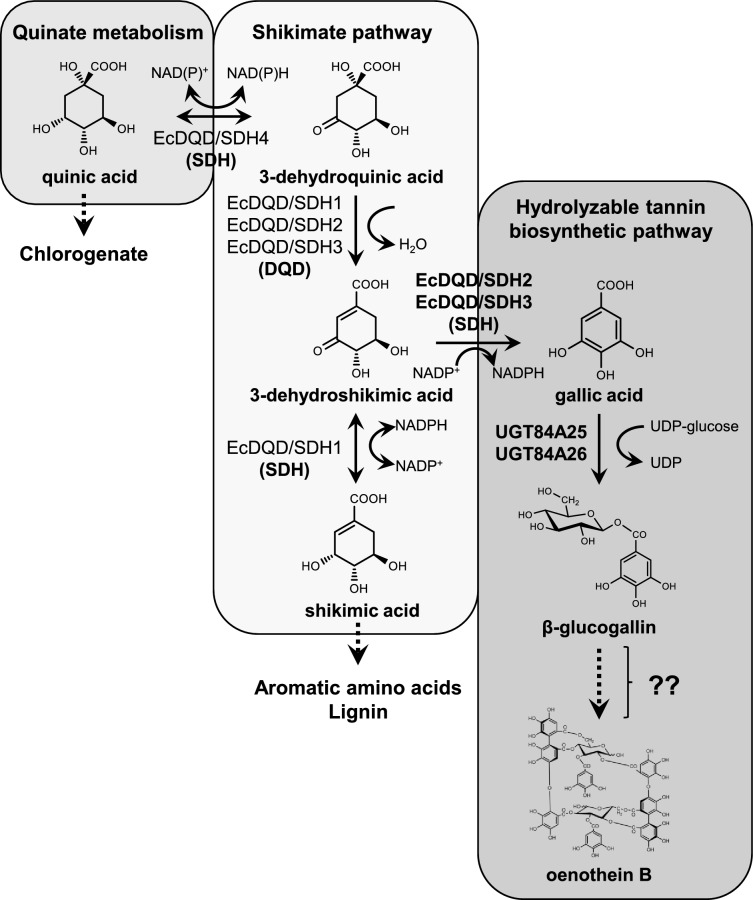
Overview of hydrolyzable tannin (HT), shikimate, and quinate biosynthesis in *E. camaldulensis*. Gallic acid biosynthesis is catalyzed by DQD/SDH family enzymes. Plant DQD/SDHs are proposed to link the shikimate pathway to the HT biosynthetic pathway. *DQD* dehydroquinate dehydratase, *SDH* shikimate dehydrogenase, *UGT* UDP-glycosyltransferase

**Fig. 2:**
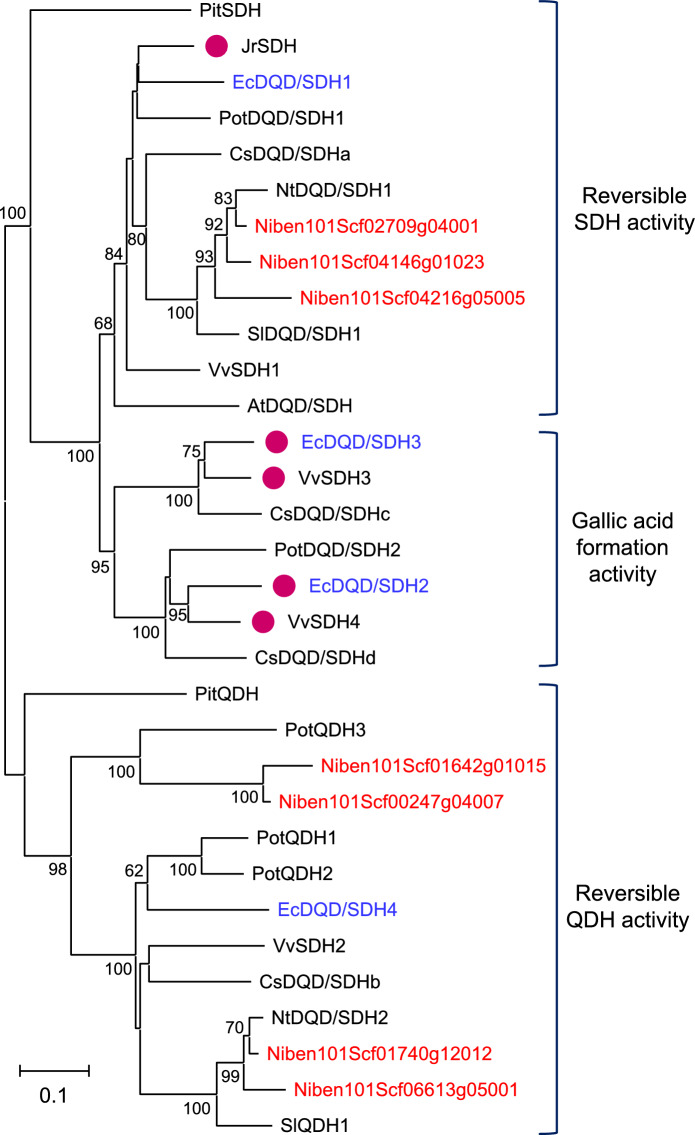
Phylogenetic analysis of *Nicotiana benthamiana* DQD/SDHs and other functionally characterized DQD/SDH family members in seed plants. The phylogenetic tree was constructed based on an alignment of multiple full-length protein sequences according to the neighbor-joining method. The scale bar represents 0.1 fixed mutations per site. Bootstrap values (1,000 replicates) greater than 60% are indicated. The DQD/SDH family members can be classified into three groups whose SDH-domains mainly exhibit the activity of reversible shikimate dehydrogenase (SDH), gallic acid formation, or reversible quinate dehydrogenase (QDH). The accession numbers of the DQD/SDHs are listed in Table S1. The DQD/SDHs from *N*. *benthamiana* and *Eucalyptus camaldulensis* are highlighted in red and blue letters, respectively. Magenta dots indicate enzymes with gallate formation activity

The HT biosynthetic pathway branches off from the shikimate pathway (Fig. 1). The shikimate pathway produces chorismate, which is an amino acid precursor, and is universally conserved in plants and microorganisms. In plants, shikimate dehydrogenase (SDH) family proteins may catalyze various reactions and link the shikimate pathway to HT and chlorogenic acid biosynthetic pathways (Bontpart et al. 2016; Guo et al. 2014). Plant SDHs form bifunctional enzymes by fusing with dehydroquinate dehydratases (DQDs), which catalyze the dehydration of 3-dehydroquinic acid to form 3-dehydroshikimic acid in the shikimate pathway (Peek and Christendat 2015). We identified four DQD/SDH family proteins (EcDQD/SDH1, 2, 3, and 4) in *E. camaldulensis* via heterologous protein production in *Escherichia coli* and in vitro catalytic activity assays (Fig. 2, Table S1). Notably, EcDQD/SDH2 and EcDQD/SDH3 catalyze the oxidation of 3-dehydroshikimic acid to gallic acid, which may link the shikimate pathway to HT biosynthesis (Fig. 1; Tahara et al. 2021). In contrast, EcDQD/SDH1 is responsible for the required reversible SDH activity in the shikimate pathway, while EcDQD/SDH4 exhibits reversible quinate dehydrogenase activity, which may link the shikimate pathway to chlorogenic acid biosynthesis. *Arabidopsis thaliana* has one DQD/SDH, which is more homologous to EcDQD/SDH1 than to the other DQD/SDHs in *E. camaldulensis*. *Nicotiana benthamiana* has seven DQD/SDHs that are homologous to EcDQD/SDH1 or 4 (Fig. 2). Thus, DQD/SDHs homologous to EcDQD/SDH2 and 3 are absent in these herbaceous model plants. Moreover, we previously identified the UDP-glycosyltransferases (UGTs) in *E. camaldulensis* (UGT84A25 and UGT84A26), which catalyze the conversion of gallic acid to β-glucogallin (1-*O*-galloyl-β-d-glucose; Figs. 1 and S1, Table S2; Tahara et al. 2018). However, the catalytic activities of these DQD/SDHs and UGTs in *E. camaldulensis* have been demonstrated by in vitro assays, but not in planta.

There is currently no report regarding the biotechnological production of β-glucogallin in HT non-accumulating plants. Accordingly, the objective of this study was to reconstitute the relevant pathway in HT non-accumulating plants. We herein describe the *Agrobacterium tumefaciens*-mediated transient expression of the *E. camaldulensis* genes *EcDQD/SDH2*, *EcDQD/SDH3*, *UGT84A25*, and *UGT84A26* in *N. benthamiana* leaves. Because of the similarities between *N. benthamiana* and other plants in terms of their cellular compartmentalization, cofactors, and coenzymes, there has been an increase in the use of *N. benthamiana* as a system for the reconstitution of the pathways associated with the production of plant natural compounds over the past decade (Reed and Osbourn 2018). The *A. tumefaciens*-mediated expression of *EcDQD/SDH2* and *EcDQD/SDH3* indicated that transgenic *N. benthamiana* leaf regions synthesized gallic acid. Furthermore, the co-expression of these four genes resulted in the production of β-glucogallin, the universal metabolic precursor of HTs. Therefore, the method established in this study may accelerate the characterization of the HT biosynthetic pathway and clarify HT functions in planta.

## Materials and methods

### Plant materials and growth conditions

*Nicotiana benthamiana* Domin plants were grown in a climate chamber under fluorescent light (16-h light/8-h dark) at 25 °C. A *Eucalyptus camaldulensis* Dehnh. clone (Myrtaceae; seed lot 19708; Australian Tree Seed Centre, CSIRO) was cultured hydroponically in a growth chamber as previously described (Tahara et al. 2018). Leaf samples were collected, immediately frozen in liquid nitrogen, and stored at − 80 °C until analyzed.

### Phylogenetic analysis

The DQD/SDH and UGT sequences of *N. benthamiana* were obtained from the draft genome v1.01 (https://solgenomics.net/). After alignment with MUSCLE, the phylogenetic analyses of DQD/SDHs and UGTs sequences were conducted with the neighbor-joining method using MEGA11 (Tamura et al. 2021).

### Taxonomic re-classification of HT distribution

Families and genera with HT-containing species determined on the basis of the Cronquist system of classification (Engelhardt et al. 2016; Okuda et al. 2000; Table S3, Fig. S2) were revised according to the Angiosperm Phylogeny Group classification (APG IV 2016).

### RNA extraction

Total RNA was extracted from *E. camaldulensis* and *N. benthamiana* leaves using the hexadecyltrimethylammonium bromide (CTAB) method and purified using the SV Total RNA Isolation System (Promega) as previously described (Tahara et al. 2018).

### Plasmid construction

**Fig. 3:**
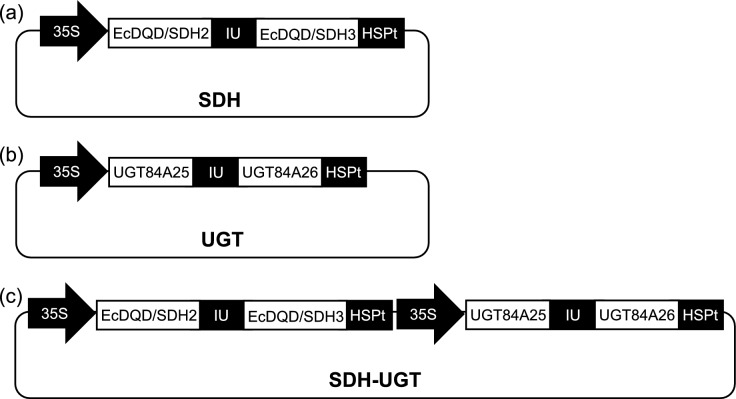
Schematic representation of the expression vectors used for the transient expression in *N. benthamiana*. **a** SDH plasmid: the cauliflower mosaic virus (CaMV) 35S promoter was fused to the *EcDQD/SDH2* and *EcDQD/SDH3* genes that sandwiched the intein-UBQ (IU) sequence. **b** UGT plasmid: the CaMV 35S promoter was fused to the *UGT84A25* and *UGT84A26* genes that sandwiched the IU sequence. **c** SDH-UGT plasmid: the expression cassettes from the SDH and UGT plasmids were ligated in tandem. After translation, the polyprotein precursor is cleaved at the N- and C-termini of the IU domain, resulting in the release of two separate proteins (Zhang et al. 2019). HSPt, heat shock protein terminator of *Arabidopsis thaliana*

First-strand cDNA was synthesized from the total RNA extracted from *E. camaldulensis* leaves and then used as the template to amplify target cDNA sequences by PCR with specific primer sets (Table S4). For transient expression of multiple proteins simultaneously in leaves of *N. benthamiana,* we employed a polycistronic expression system using intein-UBQ sequence (Zhang et al. 2019) to minimize the use of the same promoter and to achieve equal expression levels of the related enzyme genes. For the biosynthesis of gallic acid, the intein-UBQ sequence was sandwiched by the open reading frames of *EcDQD/SDH2* (Accession No. LC487989) and *EcDQD/SDH3* (LC487990) and subsequently inserted into p35SHSPstarG4_L4R1, which is the same as p35SHSPG (Oshima et al. 2011) except for the mutated *Hin*dIII site in the HSP terminator and the different Gateway *att* site (*att*L4–*att*R1) (Fig. 3a). The same method was used to prepare the construct for *UGT84A25* (LC189069) and *UGT84A26* (LC189071), which are responsible for the subsequent reaction, but the construct was inserted into p35SHSPG, which contains the Gateway *att*L1–*att*L2 site (Oshima et al. 2011; Fig. 3b). Finally, the inserted constructs in these two plasmids and the corresponding sequence in the empty vector were inserted into the pGWB501 vector (Nakagawa et al. 2009), which has the Gateway *att*R4–*att*R2 site, via a multisite Gateway reaction to prepare the recombinant plasmids for the transient expression of SDH, UGT, and SDH-UGT.

### Transient heterologous gene expression in *N. benthamiana*

The recombinant plasmids were individually introduced into *Agrobacterium tumefaciens* strain GV3101 cells harboring the transformation helper plasmid pSoup via electroporation (Hellens et al. 2000).

Overnight cultures were harvested by centrifugation and the pellets were resuspended in infiltration buffer (10 mM MgCl_2_, 10 mM MES pH 5.6, and 100 μM acetosyringone). The OD_600_ values of the suspensions were adjusted to 0.5, after which the suspensions were incubated for 2 h at room temperature prior to the infiltration of *N. benthamiana* using a needleless syringe. The infiltrated plants were cultivated in the climate chamber. At specific time-points, leaves were collected and immediately frozen in liquid nitrogen.

### Quantitative real-time PCR (RT-qPCR) analysis

PrimeScript RT Master Mix (Takara) was used to synthesize cDNA from the extracted total RNA. The RT-qPCR analysis of *EcDQD*/*SDH2*, *EcDQD*/*SDH3*, *UGT84A25*, and *UGT84A26* transcript levels was performed using TB Green Premix Ex Taq II (Takara) and the CFX96 Touch Real-Time PCR Detection System (Bio-Rad). The *N. benthamiana* gene encoding elongation factor 1-α (*NbEF-1α*) transcripts served as internal control. The gene-specific primers are listed in Table S4. The target sequences were amplified using the *E. camaldulensis* cDNA as the template and then cloned into a pBlueScript II SK ( +) vector. The *NbEF-1α* was amplified using the *N. benthamiana* cDNA as the template and then cloned into a p35SHSPG vector (Oshima et al. 2011). The plasmid solution for each gene was serially diluted tenfold (from 10^8^ to 10^3^ molecules μL^−1^) and used to generate a standard curve for the absolute quantification. To normalize against the *NbEF-1α* transcript, the relative mRNA copy number of *EcDQD/SDH2*, *EcDQD/SDH3*, *UGT84A25*, and *UGT84A26* was calculated by the following equation:

$${\text{Reltavie mRNA copy number of target gene}} = { }\frac{{\text{mRNA copy number of target gene}}}{{{\text{mRNA copy number of }}NbEF{\text{-}}1\alpha }}.$$

### Identification and quantification of HTs and related compounds

Leaf samples (1 g) were ground to a powder in liquid nitrogen using a cell disruptor (Multi-Beads Shocker, Yasui Kikai) for the subsequent extraction with 1 mL ice-cold 70% (v/v) aqueous acetone. The samples were centrifuged at 4 °C and the supernatant (50 µL) was diluted with 450 µL 0.1% (v/v) formic acid in water. Extracts were analyzed using an ultra-performance liquid chromatography (UPLC) system (ACQUITY UPLC H-Class, Waters) coupled with a quadrupole time-of-flight mass spectrometry (Q-TOF–MS) system (Xevo G2-XS QTof, Waters). The UPLC conditions were as follows: column, ACQUITY UPLC HSS T3 Column (particle size 1.8 µm, 2.1 mm × 100 mm; Waters); column temperature, 40 °C; solvent A, 0.1% (v/v) formic acid in water; solvent B, acetonitrile; flow rate, 0.5 mL min^−1^. The gradient was 0.1–25% eluent B (at 0–10 min after injection), 25–99% (10–10.1 min), 99–0.1% (12–12.1 min), and 0.1% (12.1–15 min). The Q-TOF–MS conditions were as follows: ionization mode, electrospray ionization; acquisition mode, MS^E^ or MS/MS negative-sensitivity mode; acquisition range, 40–1,200; capillary voltage, 1.0 kV; cone voltage, 30 V; source temperature, 120 °C; desolvation temperature, 500 °C; cone gas flow, 50 L h^−1^; desolvation gas flow, 1,000 L h^−1^; collision energy, 6 eV (low energy) or 10–45 eV (high energy). Compounds in the extracts were identified by comparing their retention times and product ion spectra with those of authentic standard compounds (β-glucogallin, BOC Sciences; 3-glucogallic acid and 4-glucogallic acid, Synthose). The measured mass of the compounds was consistent with their elemental composition (Table S5). Each compound was quantified according to a standard curve plotted using the peak areas in the extracted ion chromatograms of the deprotonated molecule *m*/*z* values.

### Extraction of crude protein from plants and UGT activity assay

Frozen fresh leaves (0.5 g) were mixed with 2 mL ice-cold extraction buffer (100 mM phosphate buffer pH 7.5 and 150 mM NaCl) supplemented with 0.5 g PVPP and then homogenized in a mortar. The homogenized samples were centrifuged. The supernatant was used for the overnight precipitation with ammonium sulfate (80% saturation) at 4 °C. The precipitate was suspended in 1 mL storage buffer [100 mM MES pH 5.5, 100 mM NaCl, and 10% (v/v) glycerol]. After a centrifugation at 10,000×*g* for 5 min, the supernatant was desalted using the Amicon Ultra-0.5 Centrifugal Filter Device (Merck Millipore). The protein concentrations of desalted extract were estimated using the Qubit Protein Assay Kit (Thermo Fisher Scientific) prior to the UGT activity assay.

The UGT activity assay was performed using a 100 μL reaction mixture containing 100 mM MES (pH 5.5), 4 mM UDP-glucose, 2 mM gallic acid, 3 mM 2-mercaptoethanol, and 50 μg desalted crude protein. The reaction mixture was incubated at 30 °C for 3 h. All reactions were terminated by adding an equal volume of methanol and then samples were centrifuged. The supernatant was transferred to a new tube for the UPLC-Q-TOF–MS analysis.

### Statistical analysis

The metabolite concentrations and gene expression levels are presented herein as the mean ± SE for at least three replicates. Data were analyzed using Student’s *t*-test or the Tukey–Kramer test with BellCurve for Excel version 4.04 (Social Survey Research Information).

## Results

### Distribution of HTs among flowering plants

Hydrolyzable tannins accumulate in angiosperms, but not in gymnosperms. The taxonomic classification of HT-containing plants based on the Cronquist system of classification (Okuda et al. 2000) was revised according to the Angiosperm Phylogeny Group classification (APG IV 2016, Fig. S2). The orders, families, and genera in which HTs have been detected are listed in Table S3. Most of the taxonomic orders in which HTs have been detected were revealed to comprise a particular group of angiosperms (i.e., core eudicots). *Eucalyptus camaldulensis* from the family Myrtaceae belongs to this group. Although *A. thaliana* (i.e., herbaceous model plant) also belongs to this group, there are no reports indicating it accumulates HTs. The herbaceous model plant used in this study, *N. benthamiana*, belongs to a different group (Lamiids), which does not include plant species that accumulate HTs. Exceptionally, HTs reportedly accumulate in *Nuphar japonicum*, which belongs to the order Nymphaeales, the second plant group that separated from the others after the order Amborellales.

### Transient co-expression of *E. camaldulensis DQD/SDH* and *UGT *transgenes in *N. benthamiana* leaves

**Fig. 4:**
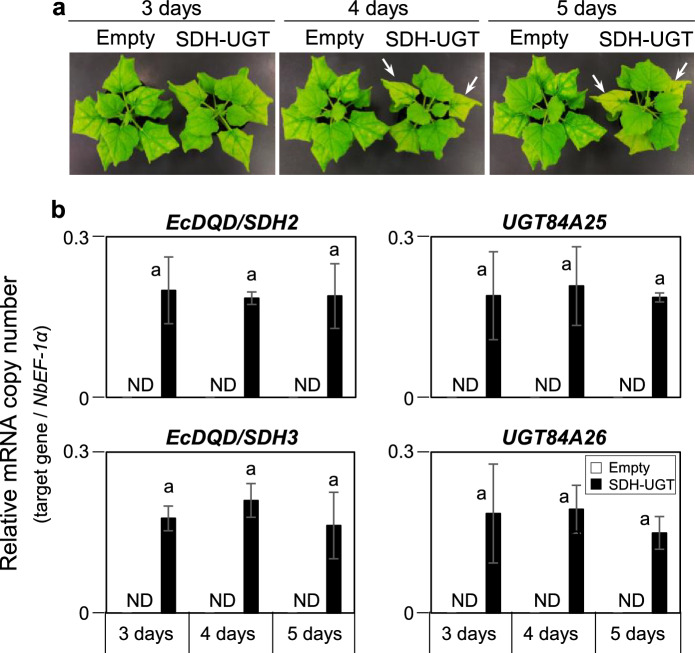
Determination of the optimal sample collection time according to a time-course analysis of transgene expression. **a** The *N. benthamiana* plants infiltrated with *A. tumefaciens* harboring the empty vector or the SDH-UGT vector are presented. The arrows indicate pale green leaves at 4 days and yellow leaves at 5 days post-infiltration. **b** RT-qPCR analyses were performed using gene-specific primers and separate standard curves. The mRNA copy numbers were normalized against the mRNA copy number of the gene encoding elongation factor 1-α (*NbEF-1α*). Data are presented as the mean ± SE (*n* = 3). *ND* not detectable. Identical letters indicate a lack of significant difference at *P* < 0.05 (Tukey–Kramer test)

During our earlier research on *E. camaldulensis*, we identified EcDQD/SDH2 and EcDQD/SDH3, which catalyze the formation of gallic acid, and UGT84A25 and UGT84A26, which catalyze the synthesis of β-glucogallin. In a specific branch of the shikimate pathway, the gallic acid produced by EcDQD/SDH2 and EcDQD/SDH3 may be further metabolized by UGT84A25 and UGT84A26 (Fig. 1). To express multiple proteins simultaneously, we constructed recombinant plasmids containing *EcDQD*/*SDH2* and *3* and/or *UGT84A25* and *26* under the control of the 35S promoter by employing the polycistronic expression system using intein-UBQ (IU) sequence (Fig. 3). The plasmids (SDH, UGT, and SDH-UGT) were then introduced into *A. tumefaciens* strain GV3101 via electroporation. First, a 5-day transgene expression analysis was performed to determine the appropriate time for sampling. *Agrobacterium tumefaciens* cells harboring the empty vector or SDH-UGT vector were used for the agroinfiltration of *N. benthamiana* leaves, which were then collected at specific time-points for the analysis of transgene expression by RT-qPCR (Fig. 4). Transgene expression was undetectable in the leaves infiltrated with *A. tumefaciens* cells containing the empty vector. The time-course analysis of the leaves infiltrated with *A. tumefaciens* cells containing the SDH-UGT vector revealed a lack of significant differences in transgene expression at 3–5 days post-infiltration (Fig. 4b). Moreover, leaves expressing SDH-UGT were pale green and yellow at 4 and 5 days post-infiltration, respectively (Fig. 4a). They were mostly necrotic at 6 days post-infiltration (Fig. S3). Thus, 3 days post-infiltration was selected as the sampling time-point.

### Expression of *E. camaldulensis DQD/SDH* and *UGT* transgenes enables the biosynthesis of the HT precursor in *N. benthamiana*

**Fig. 5:**
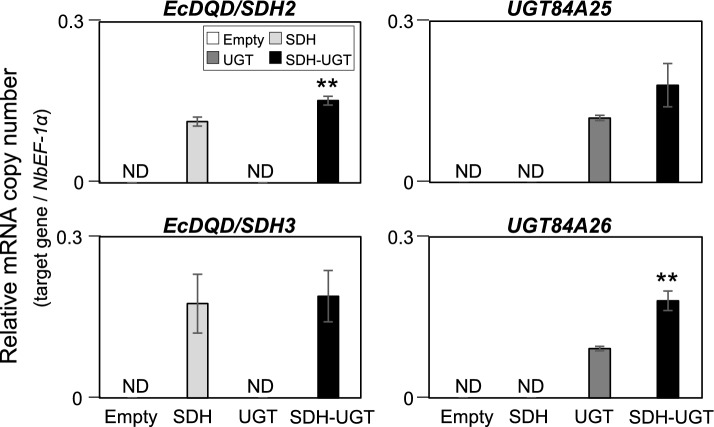
Transgene expression in leaves infiltrated with *A. tumefaciens* harboring the empty, SDH, UGT, or SDH-UGT vector. Total RNA was extracted from the leaves collected 3 days post-infiltration for the RT-qPCR analyses performed using gene-specific primers and separate standard curves. The mRNA copy numbers were normalized against the mRNA copy number of the gene encoding elongation factor 1-α (*NbEF-1α*). Data are presented as the mean ± SE (*n* = 3). ND, not detectable. Asterisks indicate a significant difference at **P < 0.01 (Student’s *t*-test) in the *EcDQD/SDH2* and *3* expression levels between the leaves expressing SDH and the leaves expressing SDH-UGT. Asterisks indicate a difference at **P < 0.01 (Student’s *t*-test) in the *UGT84A25* and *26* expression levels between the leaves expressing UGT and the leaves expressing SDH-UGT

*Agrobacterium tumefaciens* cells harboring the empty control vector or the SDH, UGT, and SDH-UGT expression vectors were used for the infiltration of *N. benthamiana* leaves. Transgene expression levels in the leaves collected 3 days later were quantified by RT-qPCR and were recorded as mRNA copy numbers relative to the *NbEF-1α* mRNA copy number (Fig. 5). Both *EcDQD/SDH2* and *EcDQD/SDH3* were expressed in the leaves infiltrated with *A. tumefaciens* harboring SDH and SDH-UGT. Similarly, *UGT84A25* and *UGT84A26* were expressed in the leaves infiltrated with *A. tumefaciens* harboring UGT and SDH-UGT.

**Fig. 6:**
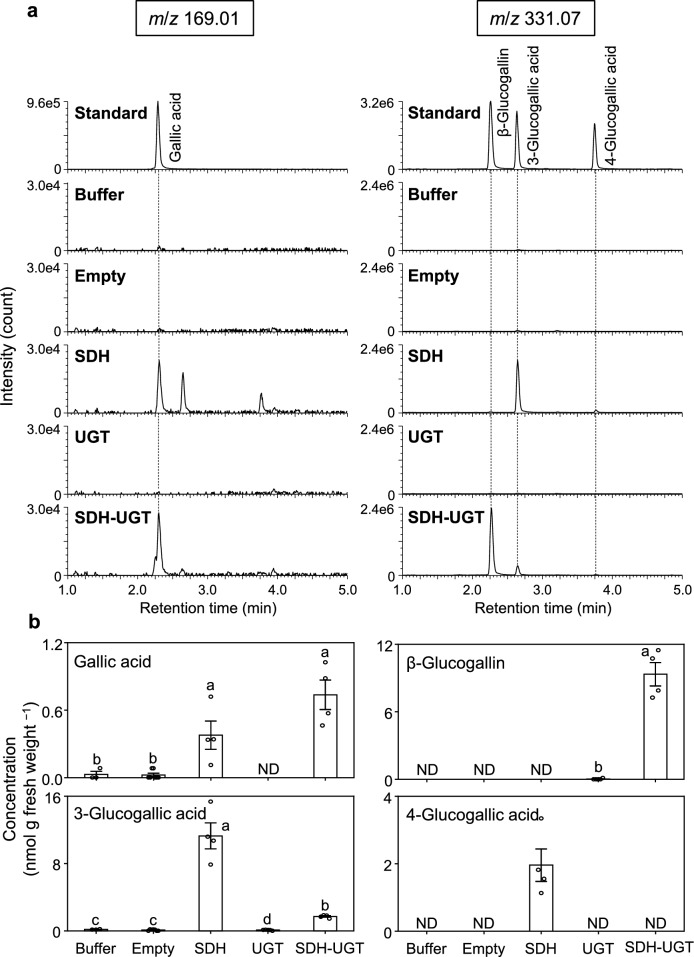
Formation of gallic acid, β-glucogallin, 3-glucogallic acid and 4-glucogallic acid in *N. benthamiana* leaves transiently expressing *E. camaldulensis DQD*/*SDH* and/or *UGT* transgenes. Leaves were collected at 3 days post-infiltration with infiltration buffer alone or *A. tumefaciens* harboring the empty, SDH, UGT, or SDH-UGT vector. **a** The UPLC-Q-TOF–MS peak profiles revealed gallic acid was formed in the leaves expressing SDH and SDH-UGT, while β-glucogallin was formed in the leaves expressing SDH-UGT. **b** Concentrations of gallic acid, β-glucogallin, 3-glucogallic acid and 4-glucogallic acid biosynthesized in transgenic *N. benthamiana* leaf regions. Data are presented as the mean ± SE (*n* = 3–7). ND, not detectable. Identical letters indicate a lack of significant difference at *P* < 0.05 (Tukey–Kramer test)

To confirm *DQD/SDH* and *UGT* genes contribute to HT biosynthesis in planta, metabolites were extracted from agroinfiltrated leaves and then analyzed by UPLC-Q-TOF–MS. The extracted ion chromatograms (*m*/*z* 169.01) for the leaves expressing SDH and SDH-UGT had an obvious peak with the same retention time as gallic acid (Fig. 6a). Furthermore, the product ion spectrum of the peak revealed a fragmentation pattern that was identical to that of gallic acid (Fig. S4). Approximately 0.3–1 nmol gallic acid was detected in 1 g leaves expressing SDH and SDH-UGT, whereas gallic acid was undetectable in the leaves infiltrated with *A. tumefaciens* harboring UGT (Fig. 6b). Unexpectedly, a very small amount of gallic acid was detected in the leaves infiltrated with buffer or *A. tumefaciens* harboring the empty vector. The extracted ion chromatograms (*m*/*z* 331.07) for the leaves expressing SDH-UGT contained an obvious peak with the same retention time as β-glucogallin (Fig. 6a). The product ion spectrum of the peak had a fragmentation pattern identical to that of β-glucogallin (Fig. S4). Approximately 10 nmol β-glucogallin was detected in 1 g leaves expressing SDH-UGT, but β-glucogallin was not detected in the leaves infiltrated with *A. tumefaciens* harboring the SDH vector or in the control leaves (Fig. 6b). The heterologous expression experiments were repeated more than 10 times, with similar results each time. The generated data suggest that the heterologous expression of *EcDQD/SDH2* and *3* as well as *UGT84A25* and *26* enables the biosynthesis of HT precursors (gallic acid and β-glucogallin) in the HT non-accumulating herbaceous model plant *N. benthamiana*. The concentrations of gallic acid and β-glucogallin produced in *N. benthamiana* were roughly one-hundredth of those in *E. camaldulensis*. In 1 g leaves of *E. camaldulensis*, 63 ± 14 nmol gallic acid and 420 ± 180 nmol β-glucogallin were detected (mean ± SD).

### Endogenous UGTs in *N. benthamiana* glucosylate gallic acid to form 3-glucogallic acid and 4-glucogallic acid

**Fig. 7:**
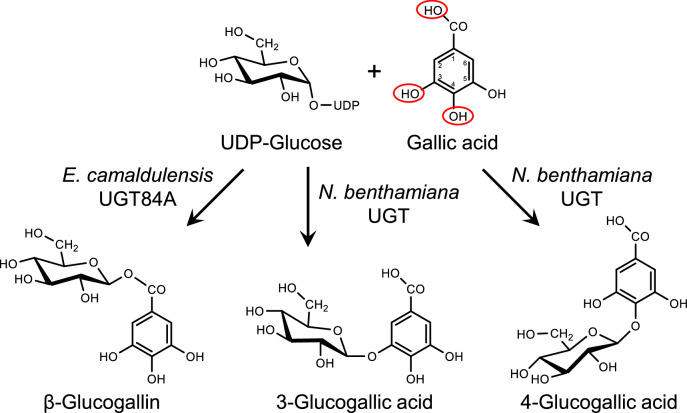
A model biosynthetic pathway for β-glucogallin, 3-glucogallic acid and 4-glucogallic acid in *N. benthamiana* leaves transiently expressing *E. camaldulensis UGT84A*s. While exogenous *E. camaldulensis* UGT84A25 and UGT84A26 catalyze the formation of β-glucogallin, endogenous *N. benthamiana* UGTs catalyze the formation of 3-glucogallic acid and 4-glucogallic acid. The position of the hydroxy group of gallic acid, to which glucose is transferred by UGT, determines which gallic acid derivative is formed

Interestingly, the extracted ion chromatograms (*m*/*z* 331.07) for the leaves expressing SDH and SDH-UGT had two obvious peaks at 2.6 and 3.8 min (Fig. 6a), implying the expression of SDH and SDH-UGT promoted the accumulation of two metabolites other than gallic acid and β-glucogallin. The deprotonated molecule peak (*m*/*z* 331.07) and the product ion peak (*m*/*z* 169.01) in the mass spectra (Fig. S4) suggested that the two metabolites have the same molecular weight as β-glucogallin and contain gallic acid as a substructure. We identified them as 3-glucogallic acid (gallic acid 3-*O*-β-D-glucoside) and 4-glucogallic acid (gallic acid 4-*O*-β-d-glucoside) by comparing their retention times and product ion spectra with those of authentic standards (Figs. 6a, 7 and S4). Approximately 10 nmol 3-glucogallic acid and 2 nmol 4-glucogallic acid were detected in 1 g leaves expressing SDH. In addition, a small amount of 3-glucogallic acid was detected in all examined leaves (Fig. 6b). These results suggest that endogenous glucosyltransferases in *N. benthamiana* catalyze the conversion of gallic acid to 3-glucogallic acid or 4-glucogallic acid.

**Fig. 8:**
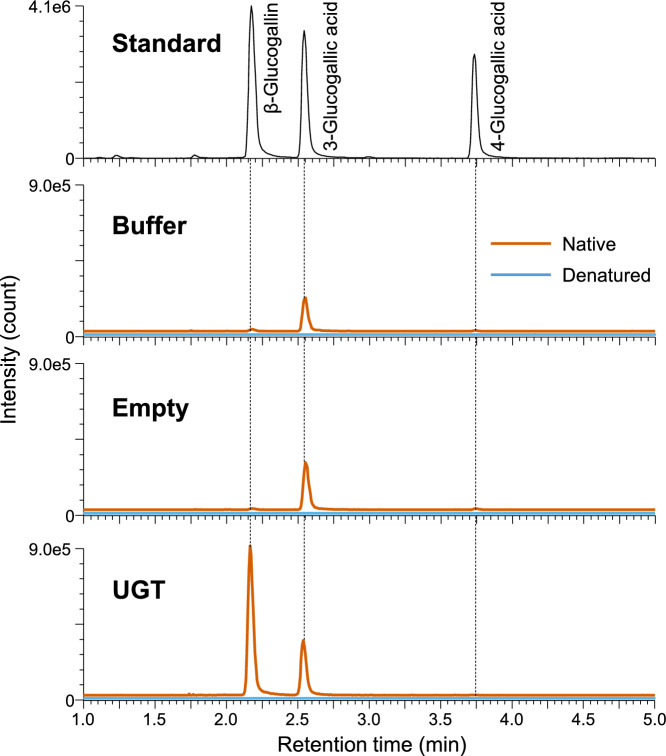
In vitro functional analysis of *E. camaldulensis* UGTs and endogenous *N. benthamiana* UGTs. Crude protein extracts from *N. benthamiana* leaves infiltrated with infiltration buffer alone or with *A. tumefaciens* harboring the empty or UGT vector were used to assay in vitro enzyme activities, with gallic acid and UDP-glucose as substrates. The UPLC-Q-TOF–MS peak profiles indicated 3-glucogallic acid and 4-glucogallic acid were formed in all assays involving native proteins. Additionally, β-glucogallin was formed in the assay involving native proteins from the leaves expressing UGT. The results of the enzyme activity assay using the native and denatured proteins are presented as peak profiles with orange and blue lines, respectively

To verify this possibility, we infiltrated *N. benthamiana* leaves with buffer or *A. tumefaciens* harboring the empty vector or the UGT expression vector. The crude protein extracts obtained from the *N. benthamiana* leaves were used for in vitro catalytic assays, with gallic acid and UDP-glucose as substrates. The reaction products were analyzed by UPLC-Q-TOF–MS, which revealed that the native crude proteins from *N. benthamiana* leaves produced 3-glucogallic acid and 4-glucogallic acid. In addition, only proteins from the *N. benthamiana* leaves expressing UGTs produced β-glucogallin (Fig. 8). All extracted ion chromatograms for the denatured proteins used in assays lacked obvious peaks. These results suggest that endogenous UDP-glucose-dependent glucosyltransferases in *N. benthamiana* catalyze the transfer of glucose to gallic acid to form 3-glucogallic acid or 4-glucogallic acid, but not β-glucogallin (Fig. 7).

### Effects of the transient expression of *E. camaldulensis SDH* and *UGT* transgenes on other metabolites

**Fig. 9:**
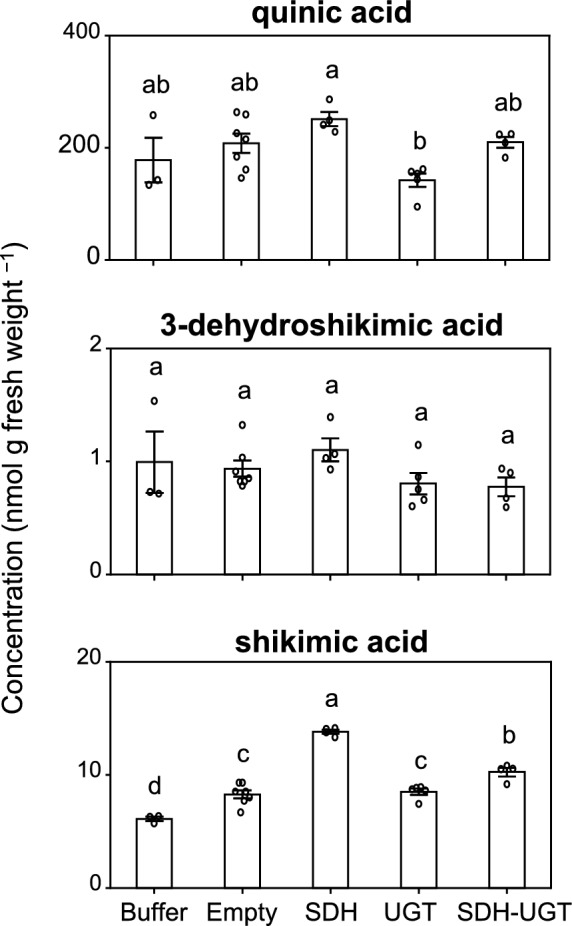
Effect of the heterologous expression of *E. camaldulensis SDH* and *UGT* transgenes on shikimic acid and quinic acid biosynthesis. The concentrations of quinic acid, 3-dehydroshikimic acid, and shikimic acid in the transgenic *N. benthamiana* leaf regions in Fig. 6 are indicated. Data are presented as the mean ± SE (*n* = 3–7). Identical letters indicate a lack of significant difference at *P* < 0.05 (Tukey–Kramer test)

To clarify how the transient expression of *E. camaldulensis DQD/SDH* and *UGT* transgenes affects other metabolites related to the shikimate pathway, metabolites extracted from agroinfiltrated leaves were analyzed by UPLC-Q-TOF–MS (Fig. 9). The transient expression of SDH, UGT, or SDH-UGT had no significant effect on the 3-dehydroshikimic acid and quinic acid concentrations. In contrast, the shikimic acid concentration increased in the leaves in which SDH was transiently expressed. The 3-dehydroquinic acid concentration was too low to quantify. These findings indicate that the yellowing of the leaves agroinfiltrated with SDH-UGT was probably due to the increased synthesis of HT precursors rather than a decrease in native metabolite contents.

## Discussion

### Demonstration of the *E. camaldulensis* DQD/SDH and UGT functions in planta

With the ultimate goal of enhancing the Al resistance of crops and trees, we attempted to identify and characterize the *E. camaldulensis* genes involved in HT biosynthesis. The heterologous protein production in *E. coli* and in vitro catalytic assays with purified enzymes showed that EcDQD/SDH2 and 3 catalyze the synthesis of gallic acid, the first intermediate in the HT biosynthetic pathway (Tahara et al. 2021). Moreover, the heterologous protein production in *E. coli* and in vitro assays demonstrated that UGT84A25 and 26 catalyze the esterification of UDP-glucose and gallic acid to form β-glucogallin, the second HT intermediate (Tahara et al. 2018). In vitro assays indicated that these enzymes (DQD/SDHs and UGTs) are important for the biosynthesis of HT precursors, but their functions in planta will need to be confirmed. *N. benthamiana* does not accumulate HTs, but pathways for several natural products, including betalains (Polturak et al. 2016) and terpenoids (Reed and Osbourn 2018), have been successfully reconstituted in this herbaceous model plant via *Agrobacterium*-mediated transient expression. In the current study, the biosynthesis of gallic acid in *N. benthamiana* leaves transiently expressing *EcDQD/SDH2* and *EcDQD/SDH3* reflected the catalytic activity of *E. camaldulensis* DQD/SDHs in planta (Figs. 6 and S4). In addition, the *A. tumefaciens*-mediated co-expression of *E. camaldulensis* UGT84A25 and UGT84A26 with DQD/SDHs resulted in the production of β-glucogallin in the transgenic *N. benthamiana* leaf regions (Figs. 6 and S4). Thus, *E. camaldulensis* UGTs can catalyze the biosynthesis of β-glucogallin in planta, with the gallic acid produced by SDH serving as a substrate.

### Successful reconstitution of part of the HT biosynthetic pathway in HT non-accumulating plants

The DQD/SDHs that catalyze the oxidation of 3-dehydroshikimic acid to produce gallic acid have also been identified in *Juglans regia* (Muir et al. 2011) and *Vitis vinifera* (Bontpart et al. 2016). Additionally, *J. regia* DQD/SDH was heterologously overexpressed in *N. tabacum*, which resulted in increased gallic acid accumulation (Muir et al. 2011). There is currently no report regarding the biotechnology-based production of the subsequent intermediate β-glucogallin in HT non-accumulating plants. In this study, the *A. tumefaciens*-mediated transient overexpression of *E. camaldulensis DQD/SDH* and *UGT* transgenes led to the formation of β-glucogallin in HT non-accumulating plants. A time-course analysis of transgene expression showed that the leaves agroinfiltrated with SDH-UGT were wilted and necrotic at the infiltration site after a long-term incubation, whereas the control leaves were normal (Figs. 4a and S3). Our unpublished study showed that the addition of 16 μM β-glucogallin to the culture medium inhibited the root elongation of *A*. *thaliana*, indicating the phytotoxicity of β-glucogallin. These observations suggest that the continuous accumulation of β-glucogallin in HT non-accumulating plants may have toxic effects. This toxicity might explain why transformed plants have not been reported to date. Transformants may be obtained using inducible vectors.

### Utility of the SDH-UGT expression system for functionally characterizing downstream candidate genes in planta

There has recently been rapid progress in the whole-genome sequencing of various plants because of the advancements in sequencing technologies, but the genetic mechanisms controlling the biosynthesis and accumulation of HTs remain relatively unknown (e.g., Wang et al. 2021). Specifically, although *DQD/SDH* and *UGT84A* family genes encoding enzymes that catalyze the synthesis of the first and second intermediates, respectively, have been identified, the genes encoding the enzymes involved in the formation of the third and higher-order intermediates remain to be identified. The third to sixth intermediates are galloylglucoses (i.e., 1,6-di-, to 1,2,3,4,6-penta-*O*-galloyl-β-d-glucoses), which are synthesized by a series of galloylations of galloylglucoses, with β-glucogallin as the galloyl donor (Niemetz and Gross 2005). Researchers speculated that serine carboxypeptidase-like acyltransferase (SCPL-AT) family proteins catalyze the galloylation reaction because the expression levels of some *SCPL-AT* homologs are consistent with the biosynthesis of galloylated flavan-3-ols in grape berries (*V. vinifera*; Bontpart et al. 2018) and persimmon fruits (*Diospyros kaki*; Akagi et al. 2009). However, nearly all of the SCPL-AT candidates will need to be functionally characterized to determine whether and how they are involved in the galloylation of metabolites in plants. Because most SCPL-ATs undergo complex post-translational processing and are secreted into organelles, it may be difficult to provide the functional proof of SCPL-ATs (Mugford and Milkowski 2012). Two SCPL-ATs (CsSCPL4 and CsSCPL5) involved in the galloylation of flavan-3-ols (epicatechin and epigallocatechin) were recently isolated from *Camellia sinensis* (Yao et al. 2022). The co-expression, post-transcriptional processing, and interactions of CsSCPL4 and CsSCPL5 in *N. benthamiana*, but not in *E. coli*, are required for their galloylation activities. In the current study, we developed a transient heterologous SDH-UGT expression system that allows HT non-accumulating *N. benthamiana* plants to accumulate β-glucogallin (i.e., HT precursor). This system may be useful for co-expressing candidate genes and elucidating the in planta functions of the encoded enzymes (e.g., SCPL-ATs) that may catalyze downstream reactions in the HT biosynthetic pathway. Hence, this system will accelerate the characterization of the HT biosynthetic pathway as well as the physiological functions of HTs in plants.

### Effect of the partial reconstitution of the HT biosynthetic pathway on related metabolites

A small amount of gallic acid was biosynthesized in all *N. benthamiana* leaves, even in the wild-type leaves (Fig. 6). This is consistent with the fact that a small amount of β-glucogallin was produced in the leaves expressing UGT (Fig. 6). In the leaves expressing SDH, gallic acid as well as 3-glucogallic acid and 4-glucogallic acid levels increased (Fig. 6). This phenomenon is due to endogenous *N. benthamiana* UGTs that catalyze the reaction that converts gallic acid to 3-glucogallic acid or 4-glucogallic acid (Figs. 7 and 8). The endogenous UGTs likely metabolize gallic acid into 3-glucogallic acid, even in the wild-type *N. benthamiana* leaves. The glucosylation by endogenous UGTs helps protect plant cells from the deleterious effects of the reactive intermediate gallic acid. In the leaves expressing SDH-UGT, three types of gallic acid derivatives with a glucose were formed (Figs. 6 and 7). The type of gallic acid derivative is determined by the position of the hydroxy group of gallic acid, to which glucose is transferred by UGT (Fig. 7). Among the three derivatives, β-glucogallin (1-*O*-β-d-galloylglucose) is an energy-rich β-acetal ester that serves as a galloyl donor during galloylation reactions, whereas 3-glucogallic acid and 4-glucogallic acid are glucosides. Although the enzymes that catalyze the conversion of gallic acid to 3-glucogallic acid have not been identified in plants, UGT72BD1 reportedly catalyzes the reaction that converts gallic acid to 4-glucogallic acid in pomegranate (*Punica granatum*) (Chang et al. 2019). Similar to UGT72BD1, some members of the UGT72 family glucosylate phenylpropanoids at the C4 position of their aromatic ring, forming 4-*O*-glucosides (Fig. S1). In the genome sequence of *N. benthamiana*, there are nine UGT72 family genes (Fig. S1), which could potentially encode UGTs responsible for 4-glucogallic acid formation in *N. benthamiana*. A comprehensive database of plant UDP-dependent glycosyltransferases (pUGTdb) has been released and will be useful for functionally annotating these enzymes (Liu et al. 2023). In the present study, shikimic acid levels were higher in the leaves overexpressing *DQD/SDH* transgenes (e.g., SDH and SDH-UGT) than in the control leaves (Fig. 9). The increase in shikimic acid may be due to the fact that EcDQD/SDH2 and EcDQD/SDH3 have shikimate-forming activities in addition to their gallate-forming activities (Tahara et al. 2021).

### Supplementary Information

Below is the link to the electronic supplementary material.

Supplementary file1 (PDF 623 kb)

## Data Availability

The data supporting the findings of this study are available on request from the corresponding author.
