# Correction: Heterologous gene expression system for the production of hydrolyzable tannin intermediates in herbaceous model plants

**DOI:** 10.1007/s10265-023-01488-y

**Published:** 2023-08-17

**Authors:** Chihiro Oda-Yamamizo, Nobutaka Mitsuda, Carsten Milkowski, Hideyuki Ito, Kentaro Ezura, Ko Tahara

**Affiliations:** 1 Department of Forest Molecular Genetics and Biotechnology, Forestry and Forest Products Research Institute (FFPRI), 1 Matsunosato, Tsukuba, 305-8687 Ibaraki Japan; 2 Bioproduction Research Institute, National Institute of Advanced Industrial Science and Technology (AIST), Tsukuba, 305-8566 Ibaraki Japan; 3 Global Zero Emission Research Center, National Institute of Advanced Industrial Science and Technology (AIST), Tsukuba, 305-8566 Ibaraki Japan; 4 AGRIPOLY: International Graduate School in Agricultural and Polymer Sciences, Martin Luther University Halle-Wittenberg, Betty-Heimann-Straße 3, 06120 Halle, Germany; 5 Faculty of Health and Welfare Science, Okayama Prefectural University, 111 Kuboki, Soja, 719- 1197 Okayama Japan; 6 Japan Society for the Promotion of Science, Tokyo, Japan

**Correction to: Journal of Plant Research**

10.1007/s10265-023-01484-2

The article Heterologous gene expression system for the production of hydrolyzable tannin intermediates in herbaceous model plants, written by Chihiro Oda Yamamizo, Nobutaka Mitsuda, Carsten Milkowski, Hideyuki Ito, Kentaro Ezura, Ko Tahara, was originally published electronically on the publisher’s internet portal on 01 August 2023 without open access. With the author(s)’ decision to opt for Open Choice the copyright of the article changed on 08 August 2023 to © The Author(s) 2023 and the article is forthwith distributed under a Creative Commons Attribution 4.0 International License, which permits use, sharing, adaptation, distribution and reproduction in any medium or format, as long as you give appropriate credit to the original author(s) and the source, provide a link to the Creative Commons licence, and indicate if changes were made. The images or other third party material in this article are included in the article’s Creative Commons licence, unless indicated otherwise in a credit line to the material. If material is not included in the article’s Creative Commons licence and your intended use is not permitted by statutory regulation or exceeds the permitted use, you will need to obtain permission directly from the copyright holder. To view a copy of this licence, visit http://creativecommons.org/licenses/by/4.0/.

The original article has been corrected.

