# Supplementary material for: Heterologous gene expression system for the production of hydrolyzable tannin intermediates in herbaceous model plants
Source: J Plant Res. 2023 Aug 1;136(6):891–905. doi: 10.1007/s10265-023-01484-2 (PMC10587339; doi:10.1007/s10265-023-01484-2)
Supplement: Supplementary file 1 — Supplementary file1 (PDF 623 kb) [file 10265_2023_1484_MOESM1_ESM.pdf]

Journal of Plant Research

Heterologous gene expression system for the production of hydrolyzable tannin intermediates in herbaceous model plants

Chihiro Oda-Yamamizo, Nobutaka Mitsuda, Carsten Milkowski, Hideyuki Ito, Kentaro Ezura, Ko Tahara

Corresponding author: Ko Tahara; Department of Forest Molecular Genetics and Biotechnology, Forestry and Forest Products Research Institute, 1 Matsunosato, Tsukuba, Ibaraki 305-8687, Japan; e-mail, taharako@affrc.go.jp

Table S1 Accession numbers of DQD/SDHs shown in Fig. 2

Table S2 Accession numbers of UGT84As and UGT72s shown in Fig. S1

Table S3 Orders, families, and genera in which hydrolyzable tannins were reportedly identified

Table S4 PCR primers used for subcloning and the quantitative real-time PCR analysis

Table S5 β-Glucogallin and related compounds identified and quantified by UPLC-Q-TOF-MS

Fig. S1 Phylogenetic analysis of UGT84As and UGT72s from *Nicotiana benthamiana* and *Arabidopsis thaliana* and their family members functionally characterized in seed plants.

Fig. S2 Distribution of hydrolyzable tannins (HTs) in flowering plants.

Fig. S3 Harmful effects of the long-term incubation of *N. benthamiana*.

Fig. S4 Product ion spectra of gallic acid, β-glucogallin, 3-glucogallic acid, and 4-glucogallic acid.

References

Table S1 Accession numbers of DQD/SDHs shown in Fig. 2

Species	Protein name	Accession	Reference
Arabidopsis thaliana (At)	DQD/SDH	AT3G06350	Singh and Christendat 2006
Camellia sinensis (Cs)	DQD/SDHa	AYP64306	Huang et al. 2019
Camellia sinensis (Cs)	DQD/SDHb	AYP64307	Huang et al. 2019
Camellia sinensis (Cs)	DQD/SDHc	AYP64308	Huang et al. 2019
Camellia sinensis (Cs)	DQD/SDHd	AYP64309	Huang et al. 2019
Eucalyptus camaldulensis (Ec)	DQD/SDH1	BBL52470	Tahara et al. 2021
Eucalyptus camaldulensis (Ec)	DQD/SDH2	BBL52471	Tahara et al. 2021
Eucalyptus camaldulensis (Ec)	DQD/SDH3	BBL52472	Tahara et al. 2021
Eucalyptus camaldulensis (Ec)	DQD/SDH4 (QDH)	BBL52473	Tahara et al. 2021
Juglans regia (Jr)	SDH	AAW65140	Muir et al. 2011
Nicotiana benthamiana (Nb)		Niben101Scf00247g04007	This study
Nicotiana benthamiana (Nb)		Niben101Scf01642g01015	This study
Nicotiana benthamiana (Nb)		Niben101Scf01740g12012	This study
Nicotiana benthamiana (Nb)		Niben101Scf02709g04001	This study
Nicotiana benthamiana (Nb)		Niben101Scf04146g01023	This study
Nicotiana benthamiana (Nb)		Niben101Scf04216g05005	This study
Nicotiana benthamiana (Nb)		Niben101Scf06613g05001	This study
Nicotiana tabacum (Nt)	DQD/SDH1	AAS90325	Ding et al. 2007
Nicotiana tabacum (Nt)	DQD/SDH2	AAS90324	Ding et al. 2007
Pinus taeda (Pit)	SDH		Carrington et al. 2018
Pinus taeda (Pit)	QDH		Carrington et al. 2018
Populus trichocarpa (Pot)	DQD/SDH1 (Poptr1)	Potri.010G019000.2	Guo et al. 2014
Populus trichocarpa (Pot)	DQD/SDH2 (Poptr5)	Potri.013G029800.1	Guo et al. 2014
Populus trichocarpa (Pot)	QDH1 (Poptr2)	Potri.013G029900.2	Guo et al. 2014
Populus trichocarpa (Pot)	QDH2 (Poptr3)	Potri.005G043400.1	Guo et al. 2014
Populus trichocarpa (Pot)	QDH3 (Poptr4)	Potri.014G135500.3	Guo et al. 2014
Solanum lycopersicum (Sl)	DQD/SDH1	AAC17991	Bischoff et al. 2001
Solanum lycopersicum (Sl)	QDH	Solyc10g038080.1.1	Gritsunov et al. 2018
Vitis vinifera (Vv)	SDH1	KU163040	Bontpart et al. 2016
Vitis vinifera (Vv)	SDH2	KU163041	Bontpart et al. 2016
Vitis vinifera (Vv)	SDH3	KU163042	Bontpart et al. 2016
Vitis vinifera (At)	SDH4	KU163043	Bontpart et al. 2016

Table S2 Accession numbers of UGT84As and UGT72s shown in Fig. S1.

Species	Protein name	Accession	Reference
Arabidopsis thaliana (At)	UGT72B1	AT4G01070	Yang et al. 2018
Arabidopsis thaliana (At)	UGT72B2	AT1G01390	This study
Arabidopsis thaliana (At)	UGT72B3	AT1G01420	Yang et al. 2018
Arabidopsis thaliana (At)	UGT72C1	AT4G36770	Yang et al. 2018
Arabidopsis thaliana (At)	UGT72D1	AT2G18570	Yang et al. 2018
Arabidopsis thaliana (At)	UGT72D2	AT2G18560	This study
Arabidopsis thaliana (At)	UGT72E1	AT3G50740	Yang et al. 2018
Arabidopsis thaliana (At)	UGT72E2	AT5G66690	Yang et al. 2018
Arabidopsis thaliana (At)	UGT72E3	AT5G26310	Yang et al. 2018
Arabidopsis thaliana (At)	UGT84A1	AT4G15480	Milkowski et al. 2000b
Arabidopsis thaliana (At)	UGT84A2	AT3G21560	Milkowski et al. 2000b
Arabidopsis thaliana (At)	UGT84A3	AT4G15490	Milkowski et al. 2000b
Arabidopsis thaliana (At)	UGT84A4	AT4G15500	Milkowski et al. 2000b
Brassica napus (Bn)	UGT84A9	AAF98390	Milkowski et al. 2000a
Brassica napus (Bn)	UGT84A10	CAJ77650	Mittasch et al. 2007
Camellia sinensis (Cs)	UGT72AM1	ASA40331	Zhao et al. 2017
Camellia sinensis (Cs)	UGT84A22	ALO19890	Cui et al. 2016
Citrus unshiu (Cu)	CitLGT (UGT84A5)	BAA93039	Kita et al. 2000
Eucalyptus camaldulensis (Ec)	UGT84A25	BBB21213	Tahara et al. 2018
Eucalyptus camaldulensis (Ec)	UGT84A26	BBB21215	Tahara et al. 2018
Fragaria × ananassa (Fa)	FaGT2 (UGT84A6)	AAU09443	Schulenburg et al. 2016
Gentiana trifloral (Gt)	GtUF6CGT1	BAQ19550	Sasaki et al. 2015
Glycine max (Gm)	UGT72X4	Glyma.08G338100	Yin et al. 2017b
Glycine max (Gm)	UGT72Z3	Glyma.08G338200	Yin et al. 2017b
Hieracium pilosella (Hp)	UGT72B11	ACB56923	Witte et al. 2009
Lotus japonicus (Lj)	UGT72V3	chr3.CM0282.950.r2.m	Yin et al. 2017a
Lotus japonicus (Lj)	UGT72Z2	LjSGA_016571	Yin et al. 2017a
Lotus japonicus (Lj)	UGT72AD1	chr1.LjT10H09.40.r2.m	Yin et al. 2017a
Lotus japonicus (Lj)	UGT72AF1	chr6.CM0118.1240.r2.d	Yin et al. 2017a
Lotus japonicus (Lj)	UGT72AH1	chr3.CM0846.120.r2.d	Yin et al. 2017a
Medicago truncatula (Mt)	UGT72L1	ACC38470	Pang et al. 2008
Nicotiana benthamiana (Nb)	UGT72B34	UHH90499	Sun et al. 2019
Nicotiana benthamiana (Nb)	UGT72B35	UHH90500	Sun et al. 2019
Nicotiana benthamiana (Nb)	UGT72AX1	UHH90503	Sun et al. 2019
Nicotiana benthamiana (Nb)	UGT72AY1	UHH90560	Sun et al. 2019

Nicotiana benthamiana (Nb)		Niben101Scf00817g06013	This study
Nicotiana benthamiana (Nb)		Niben101Scf01341g00002	This study
Nicotiana benthamiana (Nb)		Niben101Scf02565g03002	This study
Nicotiana benthamiana (Nb)		Niben101Scf03434g01013	This study
Nicotiana benthamiana (Nb)		Niben101Scf04240g00006	This study
Nicotiana benthamiana (Nb)		Niben101Scf07353g02006	This study
Nicotiana benthamiana (Nb)		Niben101Scf13710g02002	This study
Populus fremontii × *P. angustifolia* (Pfa)	UGT84A17	AII32448	Babst et al. 2014
Populus tremula × *P. alba* (Pta)	UGT72B37	QLI54353	Speeckaert et al. 2020
Populus tremula × *P. alba* (Pta)	UGT72B39	QLI54355	Speeckaert et al. 2020
Populus tremula × *P. alba* (Pta)	UGT72AZ2	QLI54351	Speeckaert et al. 2020
Punica granatum (Pg)	UGT72BD1	QHB92369	Chang et al. 2019
Punica granatum (Pg)	UGT84A23	ANN02875	Ono et al. 2016
Punica granatum (Pg)	UGT84A24	ANN02877	Ono et al. 2016
Quercus robur (Qr)	UGT84A13	AHA54051	Mittasch et al. 2014
Solanum lycopersicum (Sl)	SlUGT5	ADI33725	Louveau et al. 2011
Vitis labrusca (Vl)	VlRSgt	ABH03018	Hall and De Luca 2007
Vitis vinifera (Vv)	UGT72B27	QZP12103	Härtl et al. 2017
Vitis vinifera (Vv)	VvgGT1	AEW31187	Khater et al. 2012

Table S3 Orders, families, and genera in which hydrolyzable tannins were reportedly identified

Order	Family	Genus
Nymphaeales	Nymphaeaceae	*Nuphar*
Gunnerales	Myrothamnaceae	*Myrothamnus*[1]
Fabales	Fabaceae	*Caesalpinia, Ceratonia, Haematoxylum*
Rosales	Elaeagnaceae	*Elaeagnus, Hippophae, Shepherdia*
	Rosaceae	*Agrimonia, Filipendula, Fragaria, Geum, Rosa, Rubus, Potentilla, Sanguisorba, Sieversia*
Fagales	Betulaceae	*Alnus, Carpinus, Corylus*
	Casuarinaceae	*Casuarina*
	Fagaceae	*Castanea, Castanopsis, Quercus*
	Juglandaceae	*Juglans, Rhoiptelea*
Cucurbitales	Coriariaceae	*Coriaria*
Oxalidales	Elaeocarpaceae	*Elaeocarpus*
Malpighiales	Euphorbiaceae	*Alchornea, Aleurites, Euphorbia, Excoecaria, Macaranga, Mallotus*
	Phyllanthaceae	*Antidesma, Phyllanthus*
Geraniales	Geraniaceae	*Geranium*
Myrtales	Combretaceae	*Anogeissus, Combretum, Terminalia*
	Lythraceae	*Cuphea, Lagerstroemia, Punica, Trapa, Woodfordia*
	Melastomataceae	*Bredia, Heterocentron, Medinilla, Melastoma, Tibouchina*
	Myrtaceae	*Eucalyptus, Eugenia, Feijoa, Melaleuca, Psidium, Syzygium*
	Onagraceae	*Epilobium, Fuchsia, Oenothera*
Crossosomatales	Stachyuraceae	*Stachyurus*
Malvales	Cytinaceae	*Cytinus*
Sapindales	Aceraceae	*Acer*
	Anacardiaceae	*Cotinus, Mangifera, Rhus*
Vitales	Vitaceae	*Vitis*
Saxifragales	Altingiaceae	*Liquidambar*
	Cercidiphyllaceae	*Cercidiphyllum*
	Hamamelidaceae	*Hamamelis, Loropetalum*
	Paeoniaceae	*Paeonia*
	Saxifragaceae	*Bergenia, Tellima*
Caryophyllales	Polygonaceae	*Rheum*
	Tamaricaceae	*Reaumuria, Tamarix*
Cornales	Cornaceae	*Cornus*
	Nyssaceae	*Camptotheca*
Ericales	Ericaceae	*Arctostaphylos, Pyrola*
	Lecythidaceae	*Barringtonia*
	Theaceae	*Camellia, Gordonia, Schima*

Hydrolyzable tannin-containing genera reviewed by Okuda et al. (2000) were revised according to APG IV unless otherwise noted. [1]Engelhardt et al. (2016) reported the presence of hydrolyzable tannins in this genus.

Table S4 PCR primers used for subcloning and the quantitative real-time PCR analysis

Purpose	Gene	Forward or reverse	Sequence (5’ –3’)
Subcloning	*EcDQD/SDH2*	Fw	tacaattacagatcccATGACTCTCAGCAGCATCC
		Rev	gtcggtacccccgggACTGTTCTTCACCAATGTAT
	EcDQD/SDH3	Fw	gctgtcgaccccgggATGGGCAGCGTTCCGTTCAC
		Rev	gacttatctagttacccTGCATGTTTCTCCATGAG
	UGT84A25	Fw	ttacaattacagatcccATGGGGTCGGAGGCGCTC
		Rev	gtcggtacccccgggCGACACTACCTTCAGCTCCC
	UGT84A26	Fw	gctgtcgaccccgggATGGGGTCGGAGGCACTTGT
		Rev	ctcgtcgacggtacccccCGACACCACCTTTAACT
Real-time PCR	*EcDQD/SDH2*	Fw	TGCCATCTACACGCCAAAATTG
		Rev	CAGTTGCTTTGGTGCAGGATAC
	EcDQD/SDH3	Fw	ATGCCATTTACACACCAAAGGAC
		Rev	GTCCTGAACAATTCCTCCGGG
	UGT84A25	Fw	AGAACGCGATGAAGTGGAGC
		Rev	GCCATTGGCCGTTGACTTGC
	UGT84A26	Fw	TCCTCAGACCGGAACATCCAG
		Rev	CCATTGCCCGCCACTTTGTTG
	NbEF-1α	Fw	AGCTTTACCTCCCAAGTCATC
		Rev	CAGAACGCCTGTCAATCTTGG

Table S5 β-Glucogallin and related compounds identified and quantified by UPLC-Q-TOF-MS

Compound	Retention time (min)	Elemental composition	Measured ion	Calculated exact mass	Measured accurate mass	Error (ppm)
Quinic acid	0.66	$C_7H_{12}O_6$	$[M-H]^-$	192.0634	192.0653	9.8
Shikimic acid	0.83	$C_7H_{10}O_5$	$[M-H]^-$	174.0528	174.0531	1.6
3-Dehydroshikimic acid	1.02	$C_7H_8O_5$	$[M-H]^-$	172.0371	172.0381	5.3
β-Glucogallin	2.26	$C_{13}H_{16}O_{10}$	$[M-H]^-$	332.0744	332.0761	5.2
Gallic acid	2.29	$C_7H_6O_5$	$[M-H]^-$	170.0215	170.0240	14.7
3-Glucogallic acid	2.64	$C_{13}H_{16}O_{10}$	$[M-H]^-$	332.0744	332.0755	3.5
4-Glucogallic acid	3.75	$C_{13}H_{16}O_{10}$	$[M-H]^-$	332.0744	332.0768	7.5

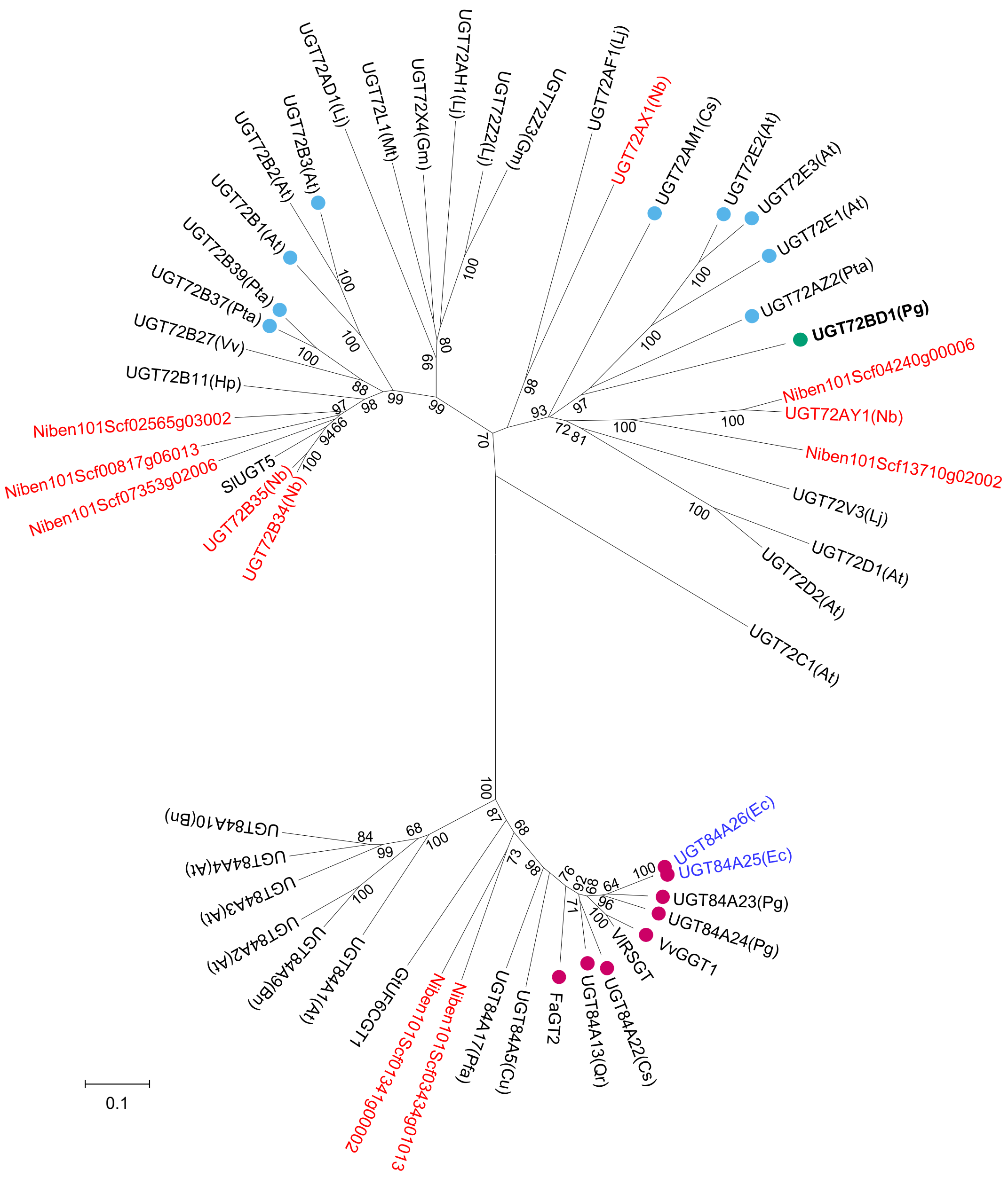

Fig. S1 Phylogenetic analysis of UGT84As and UGT72s from *Nicotiana benthamiana* and *Arabidopsis thaliana*, and their family members functionally characterized in seed plants. The phylogenetic tree was constructed based on an alignment of multiple full-length protein sequences according to the neighbor-joining method. The scale bar represents 0.1 fixed mutations per site. Bootstrap values (1,000 replicates) greater than 60% are indicated. The accession numbers of the UGTs are listed in Table S2. The UGTs from *N. benthamiana* and *Eucalyptus camaldulensis* are highlighted in red and blue letters, respectively. Magenta, green and blue dots indicate enzymes with activities of the formation of β-glucogallin, 4-glucogallic acid, and phenylpropanoid 4-*O*-glucoside, respectively.

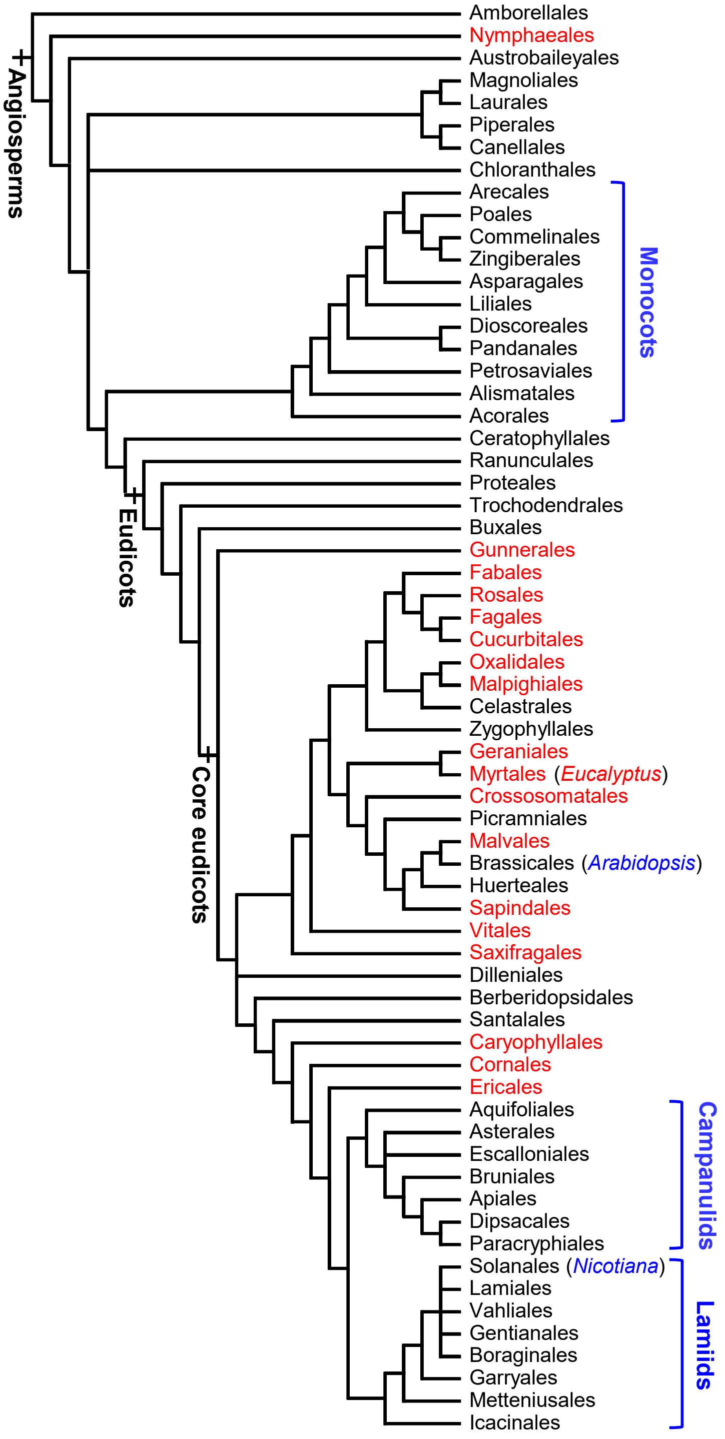

Fig. S2 Distribution of hydrolyzable tannins (HTs) in flowering plants. Orders in which HTs were reportedly identified are indicated in red in the phylogenetic tree constructed based on the Angiosperm Phylogeny Group classification (APG IV). Families and genera in which HTs were reportedly identified are listed in Table S3.

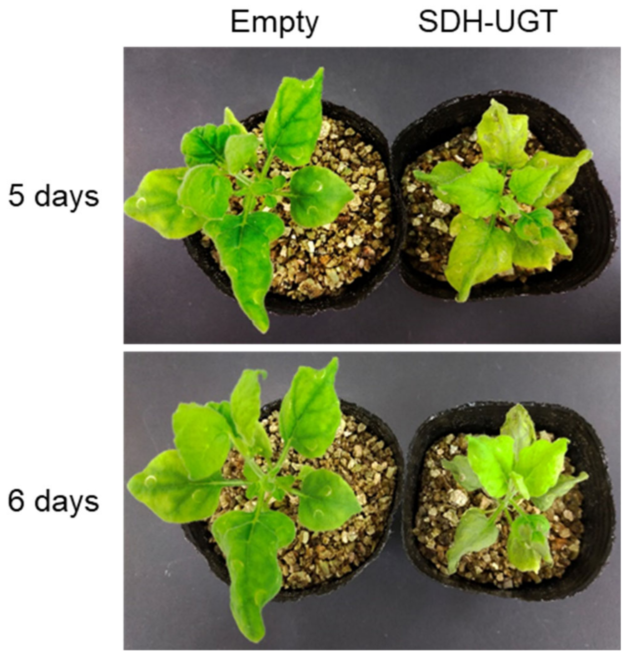

Fig. S3 Harmful effects of the long-term incubation of *N. benthamiana*. The *N. benthamiana* plants infiltrated with *A. tumefaciens* harboring the empty vector or the SDH-UGT vector are presented.

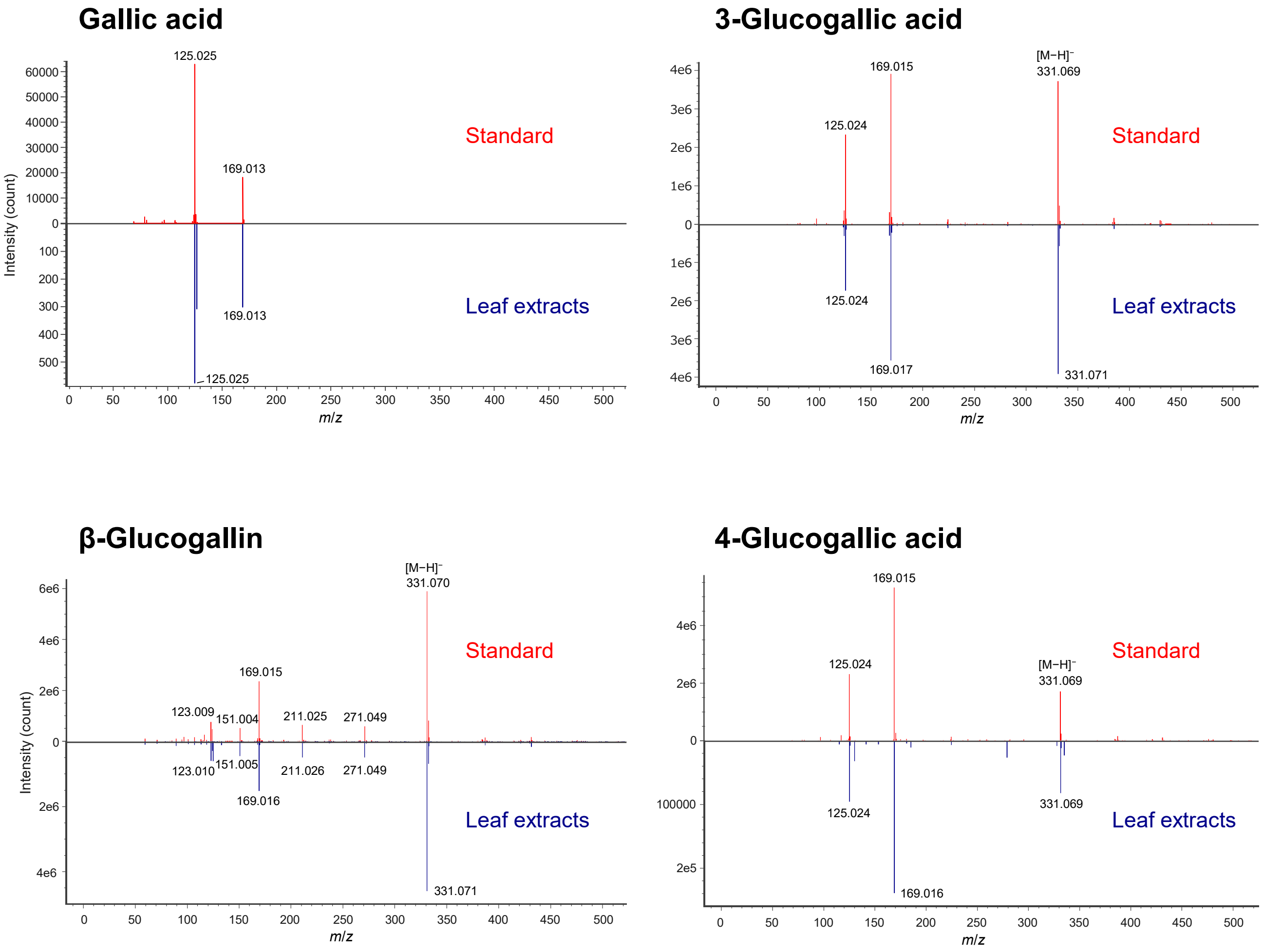

Fig. S4 Product ion spectra of gallic acid, β-glucogallin, 3-glucogallic acid, and 4-glucogallic acid. Product ion spectra were acquired at high collision energies (10–45 eV) using the UPLC-Q-TOF-MS system and then compared between authentic standards and leaf extracts.

References

Babst BA, Chen HY, Wang HQ, Payyavula RS, Thomas TP, Harding SA, Tsai CJ (2014) Stress-responsive hydroxycinnamate glycosyltransferase modulates phenylpropanoid metabolism in *Populus*. J Exp Bot 65:4191–4200. https://doi.org/10.1093/jxb/eru192

Bischoff M, Schaller A, Bieri F, Kessler F, Amrhein N, Schmid J (2001) Molecular characterization of tomato 3-dehydroquinate dehydratase-shikimate:NADP oxidoreductase. Plant Physiol 125:1891–1900. https://doi.org/10.1104/pp.125.4.1891

Bontpart T, Marlin T, Vialet S, Guiraud JL, Pinasseau L, Meudec E, Sommerer N, Cheynier V, Terrier N (2016) Two shikimate dehydrogenases, VvSDH3 and VvSDH4, are involved in gallic acid biosynthesis in grapevine. J Exp Bot 67:3537–3550. https://doi.org/10.1093/jxb/erw184

Carrington Y, Guo J, Le CH, Fillo A, Kwon J, Tran LT, Ehlting J (2018) Evolution of a secondary metabolic pathway from primary metabolism: shikimate and quinate biosynthesis in plants. Plant J 95:823–833. https://doi.org/10.1111/tpj.13990

Chang L, Wu S, Tian L (2019) Effective genome editing and identification of a regiospecific gallic acid 4-*O*-glycosyltransferase in pomegranate (*Punica granatum* L.). Hort Res 6:123. https://doi.org/10.1038/s41438-019-0206-7

Cui L, Yao S, Dai X, Yin Q, Liu Y, Jiang X, Wu Y, Qian Y, Pang Y, Gao L, Xia T (2016) Identification of UDP-glycosyltransferases involved in the biosynthesis of astringent taste compounds in tea (*Camellia sinensis*). J Exp Bot 67:2285–2297. https://doi.org/10.1093/jxb/erw053

Ding L, Hofius D, Hajirezaei M-R, Fernie AR, Börnke F, Sonnewald U (2007) Functional analysis of the essential bifunctional tobacco enzyme 3-dehydroquinate dehydratase/shikimate dehydrogenase in transgenic tobacco plants. J Exp Bot 58:2053–2067. https://doi.org/10.1093/jxb/erm059

Engelhardt C, Petereit F, Lechtenberg M, Liefländer-Wulf U, Hensel A (2016) Qualitative and quantitative phytochemical characterization of Myrothamnus flabellifolia Welw. Fitoterapia 114:69–80. https://doi.org/10.1016/j.fitote.2016.08.013

Gritsunov A, Peek J, Caballero JD, Guttman D, Christendat D (2018) Structural and biochemical approaches uncover multiple evolutionary trajectories of plant quinate dehydrogenases. Plant J 95:812–822. https://doi.org/10.1111/tpj.13989

Guo J, Carrington Y, Alber A, Ehlting J (2014) Molecular characterization of quinate and shikimate metabolism in *Populus trichocarpa*. J Biol Chem 289:23846–23858. https://doi.org/10.1074/jbc.M114.558536

Hall D, De Luca V (2007) Mesocarp localization of a bi-functional resveratrol/hydroxycinnamic acid glucosyltransferase of Concord grape (*Vitis labrusca*). Plant J 49:579–591. https://doi.org/10.1111/j.1365-313X.2006.02987.x

Härtl K, Huang FC, Giri AP, Franz-Oberdorf K, Frotscher J, Shao Y, Hoffmann T, Schwab W (2017) Glucosylation of smoke-derived volatiles in grapevine (*Vitis vinifera*) is catalyzed by a promiscuous resveratrol/guaiacol glucosyltransferase. J Agric Food Chem 65:5681–5689. https://doi.org/10.1021/acs.jafc.7b01886

Khater F, Fournand D, Vialet S, Meudec E, Cheynier V, Terrier N (2012) Identification and functional characterization of cDNAs coding for hydroxybenzoate/hydroxycinnamate glucosyltransferases co-expressed with genes related to proanthocyanidin biosynthesis. J Exp Bot 63:1201–1214. https://doi.org/10.1093/jxb/err340

Kita M, Hirata Y, Moriguchi T, Endo-Inagaki T, Matsumoto R, Hasegawa S, Suhayda CG, Omura M (2000) Molecular cloning and characterization of a novel gene encoding limonoid UDP-glucosyltransferase in *Citrus*. FEBS Lett 469:173–178. https://doi.org/10.1016/S0014-5793(00)01275-8

Huang K, Li M, Liu Y, Zhu M, Zhao G, Zhou Y, Zhang L, Wu Y, Dai X, Xia T, Gao L (2019) Functional analysis of 3-dehydroquinate dehydratase/shikimate dehydrogenases involved in shikimate pathway in *Camellia sinensis*. Front Plant Sci 10:1268. https://doi.org/10.3389/fpls.2019.01268

Louveau T, Leitao C, Green S, Hamiaux C, van der Rest B, Dechy-Cabaret O, Atkinson RG, Chervin C (2011) Predicting the substrate specificity of a glycosyltransferase implicated in the production of phenolic volatiles in tomato fruit. FEBS J 278:390–400. https://doi.org/10.1111/j.1742-4658.2010.07962.x

Milkowski C, Baumert A, Strack D (2000a) Cloning and heterologous expression of a rape cDNA encoding UDP-glucose : sinapate glucosyltransferase. Planta 211:883–886. https://doi.org/10.1007/s004250000411

Milkowski C, Baumert A, Strack D (2000b) Identification of four *Arabidopsis* genes encoding hydroxycinnamate glucosyltransferases. FEBS Lett 486:183–184. https://doi.org/10.1016/S0014-5793(00)02270-5

Mittasch J, Böttcher C, Frolova N, Bönn M, Milkowski C (2014) Identification of UGT84A13 as a candidate enzyme

for the first committed step of gallotannin biosynthesis in pedunculate oak (*Quercus robur*). Phytochemistry 99:44–51. https://doi.org/10.1016/j.phytochem.2013.11.023

Mittasch J, Strack D, Milkowski C (2007) Secondary product glycosyltransferases in seeds of *Brassica napus*. Planta 225:515–522. https://doi.org/10.1007/s00425-006-0360-7

Muir RM, Ibanez AM, Uratsu SL, Ingham ES, Leslie CA, McGranahan GH, Batra N, Goyal S, Joseph J, Jemmis ED, Dandekar AM (2011) Mechanism of gallic acid biosynthesis in bacteria (*Escherichia coli*) and walnut (*Juglans regia*). Plant Mol Biol 75:555–565. https://doi.org/10.1007/s11103-011-9739-3

Okuda T, Yoshida T, Hatano T (2000) Correlation of oxidative transformations of hydrolysable tannins and plant evolution. Phytochemistry 55: 513–529. https://doi.org/10.1016/S0031-9422(00)00232-6

Ono NN, Qin XQ, Wilson AE, Li G, Tian L (2016) Two UGT84 family glycosyltransferases catalyze a critical reaction of hydrolyzable tannin biosynthesis in pomegranate (*Punica granatum*). PLoS ONE 11:e0156319. https://doi.org/10.1371/journal.pone.0156319

Pang Y, Peel GJ, Sharma SB, Tang Y, Dixon RA (2008) A transcript profiling approach reveals an epicatechin-specific glucosyltransferase expressed in the seed coat of *Medicago truncatula*. Proc Natl Acad Sci USA 105:14210–14215. https://doi.org/10.1073/pnas.0805954105

Sasaki N, Nishizaki Y, Yamada E, Tatsuzawa F, Nakatsuka T, Takahashi H, Nishihara M (2015) Identification of the glucosyltransferase that mediates direct flavone *C*-glucosylation in *Gentiana triflora*. FEBS Lett 589:182–187. https://doi.org/10.1016/j.febslet.2014.11.045

Schulenburg K, Feller A, Hoffmann T, Schecker JH, Martens S, Schwab W (2016) Formation of β-glucogallin, the precursor of ellagic acid in strawberry and raspberry. J Exp Bot 67:2299–2308. https://doi.org/10.1093/jxb/erw036

Singh SA, Christendat D (2006) Structure of Arabidopsis dehydroquinate dehydratase-shikimate dehydrogenase and implications for metabolic channeling in the shikimate pathway. Biochemistry 45:7787–7796. https://doi.org/10.1021/bi060366+

Speeckaert N, Adamou NM, Hassane HA, Baldacci-Cresp F, Mol A, Goeminne G, Boerjan W, Duez P, Hawkins S, Neutelings G, Hoffmann T, Schwab W, El Jaziri M, Behr M, Baucher M (2020) Characterization of the UDP-glycosyltransferase UGT72 family in poplar and identification of genes involved in the glycosylation of monolignols. Int J Mol Sci 21. https://doi.org/10.3390/ijms21145018

Sun G, Strebl M, Merz M, Blamberg R, Huang FC, McGraphery K, Hoffmann T, Schwab W (2019) Glucosylation of the phytoalexin *N*-feruloyl tyramine modulates the levels of pathogen-responsive metabolites in *Nicotiana benthamiana*. Plant J 100:20–37. https://doi.org/10.1111/tpj.14420

Tahara K, Nishiguchi M, Frolov A, Mittasch J, Milkowski C (2018) Identification of UDP glucosyltransferase from the aluminum-resistance tree *Eucalyptus camaldulensis* forming β-glucogallin, the precursor of hydrolyzable tannins. Phytochemistry 152:154–161. https://doi.org/10.1016/j.phytochem.2018.05.005

Tahara K, Nishiguchi M, Funke E, Miyazawa S, Miyama T, Milkowski C (2021) Dehydroquinate dehydratase/shikimate dehydrogenases involved in gallate biosynthesis of the aluminum-tolerant tree species Eucalyptus camaldulensis. Planta 253:3. https://doi.org/10.1007/s00425-020-03516-w

Witte S, Moco S, Vervoort J, Matern U, Martens S (2009) Recombinant expression and functional characterisation of regiospecific flavonoid glucosyltransferases from *Hieracium pilosella* L. Planta 229:1135–1146. https://doi.org/10.1007/s00425-009-0902-x

Yang M, Fehl C, Lees KV, Lim E-K, Offen WA, Davies GJ, Bowles DJ, Davidson MG, Roberts SJ, Davis BG (2018) Functional and informatics analysis enables glycosyltransferase activity prediction. Nat Chem Biol 14:1109–1117. https://doi.org/10.1038/s41589-018-0154-9

Yin QG, Shen GA, Chang ZZ, Tang YH, Gao HW, Pang YZ (2017) Involvement of three putative glucosyltransferases from the UGT72 family in flavonol glucoside/rhamnoside biosynthesis in *Lotus japonicus* seeds. J Exp Bot 68:594–609. https://doi.org/10.1093/jxb/erw420

Yin QG, Shen GA, Di SK, Fan CY, Chang ZZ, Pang YZ (2017) Genome-wide identification and functional characterization of UDP-glucosyltransferase genes involved in flavonoid biosynthesis in *Glycine max*. Plant Cell Physiol 58:1558–1572. https://doi.org/10.1093/pcp/pcx081

Zhao X, Dai X, Gao L, Guo L, Zhuang J, Liu Y, Ma X, Wang R, Xia T, Wang Y (2017) Functional analysis of an uridine diphosphate glycosyltransferase involved in the biosynthesis of polyphenolic glucoside in tea plants (*Camellia sinensis*). J Agric Food Chem 65:10993–11001. https://doi.org/10.1021/acs.jafc.7b04969
